# Autobiography of the Insane

**Published:** 1863-04

**Authors:** 


### Art. XIII.—AUTOBIOGRAPHY OF THE INSANE.

In some of our previous volumes we have placed upon record several curious and highly-interesting narratives proceeding from the pens of persons who have passed through the varied phases of unhealthy and insane thought, descriptive of the actual state of the mind when under the influence of disease. In further illustration of this department of psychological medicine, we now place before our readers the following remarkable confession, extracted from a recent number of a valued contemporary—viz., *The American Journal of Insanity*.

"I was born April 9th, 1840, and am just nineteen. My health is good, and constitution strong, I think, though as a child I was delicate, owing to over-study. My temperament is melancholy, though not gloomy. I seldom if ever suffer from what people call 'the blues.' My mother's uncle drove himself mad trying to solve the problem of perpetual motion. My father never exhibited any peculiarities of mind, or saw visions, until his last illness. He always had a presentiment that he should die when about forty-three or forty-four years of age. He was not superstitious, but always laughed at my visions as fancies.

"The first time I was alarmed by an apparition was, I think, in 1855, when I was on a visit to Ireland. One day I was preparing to attend a party, and had gone to my room early in the afternoon to lay out my clothes ready for the evening; also to sew some rosettes on my shoes, in which I was engaged just in front of the looking-glass, where, glancing up, I saw reflected a face with grey hair, looking over my shoulder. I was not afraid, but thought, 'How foolish I am;' worked on a little, and looked up again. It was still there. Trying to believe I had been deceived, I worked on for a few seconds, and then looked again—and there it was! Thoroughly frightened, I ran from the room, not to re-enter it alone. Next day I wrote home to ask if anything was the matter. They answered me that all were well but my uncle, who had been very ill, *but was better then.* When a month later I returned, I learnt that he had died just about the time I had seen the face in the glass, but that they did not like to tell me, for fear of spoiling my pleasure. When I returned to England, the brother of the young lady whom I went to visit came to stay at our house for a time. He was a fine youth of twenty, with very large and peculiarly earnest hazel eyes, very curly hair, and altogether of very unmistakeable appearance. He remained with us for about two months, when having an appointment in India, he left us. Arrived there he wrote home regularly, saying that he liked the place *so* much, that it agreed with him *so* well, and that he was never better in his life.

"One morning, either two or three days after what we call 'Guy Fawkes' day' (5th day of November), I woke suddenly—with all my senses perfectly clear, which was the more strange as I had ever been most difficult to arouse. The moment I opened my eyes, I saw my friend George B., bending over me, his face within a few inches of mine, his eyes so fixed into mine that I could not withdraw my gaze. It was broad daylight, being about eight or after, and I saw he was in his usual dress, and, even to the curls of his hair, looking as distinct in form and colour as a living figure. Much surprised, though not in the least frightened, but, on the contrary, experiencing a most unearthly calmness (as I always do when I see these visions), I arose to a sitting posture. He also arose till he stood upright, and still looking earnestly at me, he receded a few steps, then disappeared. I did not feel alarmed, but got up and dressed at once, for fear that when I told my friends they should say it was all a dream. All that day, wherever I went, a ceaseless knocking followed me; and, though our house was very large, I heard it in every part. If I went into my dressing-room, I heard it there on the toilet-table, and in the drawing and dining-rooms, though each on different stories. Going through the halls and passages, it rapped along the walls. In fact, I heard it everywhere, except in the streets. My friends laughed at me when I said I was sure I should hear some evil of George.

"A day or so after, I went from my own room to sleep in that of a young lady who was staying with us at the time. It was a large double-bedded room, and the night was bright and moon-light. The candle had been out some little time, and my friend was asleep, as I could hear by her heavy, regular breathing. Suddenly I saw a tall white figure near the door at the foot of the bed. It walked right up on it, and came close to me. Thinking it was Miss B. walking in her sleep, I sprang up, saying, 'Miss B.! Oh, Miss B., where are you going?' at the same time trying to clasp her. My arms went through the figure, and then I knew it was no mortal. Somewhat frightened now, I cowered down, and ere long fell asleep, more than ever convinced that my friend George was either dead or dying. Very soon afterwards we heard that he had died of fever on the 8th of November, the date of the first appearance.

"This happened in November, 1855, and the following May we came to Canada, and settled in G——. One evening, papa, mamma, sister, and myself were invited to the house of an acquaintance to spend a social evening, with cards, music, &c. Not feeling inclined to join the card-players, I sat down at the piano, feeling unaccountably sad. The door was just, or rather nearly opposite to me, being on the left of the piano. Of a sudden I looked up, and was astonished to see poor George B. standing in the doorway, the lights shining full on him, and he looking earnestly at me. Thinking I had deceived myself, I played a little, and looked up. Yes, there he was, without doubt. I turned away, played on, then looked again; still he was there. Calling my sister, I asked her to go into the hall with me. We went. Not a soul—or rather nobody—had been near the place. I told mamma of the occurrence, and when we looked to see the day of the month, we found that it was the 8th of November.

"The next time that I saw anything of the kind was just before we left G——, to come to T——. I had gone into the kitchen for something. The girl was in the garden, and I distinctly saw a woman standing in the doorway. A few evenings afterwards, we were all sitting around the supper-table, on which burned two large spirit-lamps, when I saw a woman, dressed in black, standing behind papa's chair. Leaning on it, the light fell full on her. She was a stranger to me, and bore no resemblance to any one I knew. I did not at the time, but do now think it was a warning of my papa's death. I told him, and as usual he laughed at me. I saw nothing more till just before my papa's and sister's illness. My health was delicate at the time, owing perhaps to change of climate. We were at this time in T——, and residing in Ann-street. One evening, feeling tired, I left the rest of the family at supper, and came to bed by myself. In passing my dressing-room, on the way to my bed-room, I saw a head looking out on me from behind the door. I called out to them to come quickly up, as I was lonely or ill, or some such excuse, I forget what, but I did not say a word of what I had seen, not liking to make the rest nervous. A few days after this, I was in my dressing-room. It was in the afternoon, about two o'clock, perhaps. I stood in front of the looking-glass arranging my hair, when I saw reflected a bright, fresh, rosy-looking face—just such a face as my poor sister's. I turned round and heard at the same time, and for a quarter of a minute and more after, a sound resembling the dropping of a number of pieces of tin, or silver coins, all over the floor of the room. Greatly surprised, I told papa at once; also what I had seen a few nights previously.

"Not long after this, and on the very night-week before that on which papa was taken ill, we were all invited to spend the evening at the house of a friend. Mamma was too ill to go; and partly because I was fatigued, and partly to keep her company, I determined, though nearly dressed, to stay at home. So papa and my sisters went. I took a book and sat down at the table to read, as mamma soon feel asleep. Our girl went to bed about nine, and I was the only one in the house awake. I was so deeply interested in my book that I did not notice how time passed. Presently I heard some one, with, judging from the sound, very long nails beating on the table. Looking up, I saw seated opposite me, so close that by stretching out my hand I could have touched him, a man in ordinary black clothes. He was on the chair, at the foot of mamma's couch. Directly I looked up the nails ceased tapping the table. As I looked at him he vanished. I saw him for about four seconds, I should think. You may fancy I was neither nervous nor excited, when I tell you I did not disturb mamma, but sat there for three or four hours longer, till papa came in. I own I was shocked, but not nervous or excited. Papa was surprised and grieved to see me looking so ill when he came, and attributed it to being up too late. Not wishing to frighten mamma, I said nothing about the vision till next day, when papa, anxious to dispel my fears, said, 'Why, you silly child, what nonsense! Here am I, strong and well, and yet a night or two since, when I went to bed I saw opposite me a bed, myself lying dead on it; and every time I opened my eyes I saw the same.' Within a week from this he was taken ill, and died in a few weeks. During the last week of his illness scarcely a night passed but I saw some apparition. The first time I was disturbed was just about a week before his death. I was lying awake, not at all nervous, for I had not the least idea that I should lose my papa. My face was turned to the wall, when I felt the pressure as of a heavy hand on the pillow behind me. Ice-cold fingers touched me, and a cold hand encircled my neck. Such horror seized me that I must have become insensible, for sense and recollection left me. Next morning I mentioned this to mamma. All that week, to the time of papa's death, I saw women in white, and sometimes in black, at my bedside. What was very strange, too, all the night that poor papa was dying, I saw two women in the room, besides mamma and the nurse. When I entered, or looked up from papa, who required our unceasing care, I saw a strange woman in black standing behind nurse, and another at the door. After his death I saw no more of them, at least not till my sister was seriously ill. She at the time of papa's death was poorly with influenza, nothing serious. She had taken a powder to induce perspiration the previous night, but hearing about seven next morning, from our cries, that papa was going, she rushed from her bed without throwing anything round her, and kissed him just as he breathed his last sigh. Then she refused to go to bed again, threw herself down on the rug in the parlour, with her head to the fire, where she persisted in lying, and kept calling for brandy and water, which was foolishly brought her by the servant and nurse, we being too distracted to notice anything. The consequence was, she became feverish, and was obliged to take to her bed. In the meantime, I bore up as well as I could, feeling that as eldest child I should not give way, but endeavour to comfort the others, and poor mamma; so till night I never shed a tear, but went in with every one who called to where papa lay. But in the evening I could not restrain myself any longer, and had hysterics. On one of these occasions a gentleman friend carried me fainting into the street for air. It was very quiet, when suddenly we both heard a loud voice, coming from we could not tell where, and saying in distressed and agonized tones, 'Fanny,' 'Fanny,' 'Fanny!' as much as to say, 'Oh, do not, I entreat you, distress yourself so!' In a moment I was calm and strong. We neither of us said a word about the voice, but entered the house at once. Next day he asked me if I had heard it. I told him I had, and, seeing that the thought greatly agitated me, he added, 'Oh, I dare say it was some one calling Harry!" but I knew better, for nothing could be more distinct than the voice and words. A day or two afterwards, I went to my sister's room to sit with her, as she was lonely. It was about seven in the evening. As I ascended the stairs with a lamp in my hand, I saw two women robed in black at the top, one each side of the stairway. I was suffering too deeply to feel fear, so went on. The figures disappeared as I neared them. As I entered the room where my sister lay, I saw papa behind the door, looking very pale. I looked several times to make sure I had not been deceived, and each time saw him there. I sat down on the bed with my back towards the figure, until I could bear it no longer, when I called some one else to take my place, for I knew no one else in the house could see the spectre. I think it was the next day the doctors said we must all leave the house at once, or we too should have the fever; so we went to the house of a friend.

"One evening, a few days after my arrival, a loud ring at the door bell woke me. I started up, and saw, as I imagined, one of the ladies of the house by my side. I spoke to the figure, and it vanished; and at the same time I heard my friends saying something about 'poor Sophia,' my sister's name. Greatly alarmed, I called to them to bring a light, as I was sure I had seen some one in my room. I then asked who it was that rang at that early hour (about four or five o'clock). They told me it was one sent out to say that there was a change in my sister. I thought they meant a favourable change, so fell asleep, feeling happier and more hopeful than I had felt since papa's death. The same day my friends broke the tidings of my sister's death to me as gently as possible. It had taken place about three o'clock in the morning, and mamma had at once sent to acquaint us with the melancholy intelligence.

"From that time till last May I saw nothing. Last Queen's birthday I had been out, walking about with a gentleman friend. Towards evening we came in, and I went to my room to change my walking-dress. I had nearly finished dressing, and had only to get on my slippers, when, turning round, I saw papa standing near the door. So distinct was it that I felt frightened, and, snatching up the lamp, I rushed from the room. When I reached the parlour where they were all sitting, I felt reassured and somewhat ashamed; and as in my hurry I had forgotten my slippers, I determined to return for them. So taking the lamp, I opened the folding-doors between the front and back parlours, and ran up against the figure. I met no resisting power; had I done so, I should have hurt myself severely, no doubt.

I was greatly agitated when I saw it, and rushed back to mamma, who inquired what was the matter with me, I looked so ill. I told her what I had seen.

"One night, some months after this, a gentleman friend called. He had not been long present before I had occasion to go up-stairs for something. I did not take a lamp, not being afraid, but went in the dark. Coming down, just as I reached the bottom of the stairs, I saw papa standing within a foot or two of me. A soft phosphoric radiance seemed to surround him. He was very pale, as I saw distinctly by the strange light, though all was dark around me. I was very much frightened, as I should have to pass close to him to re-enter the parlour. My brain seemed to reel as I ran desperately past and gained the room where they were all sitting. When I told them how I had been alarmed, some one went into the passage, but saw nothing.

"The last and by far the most horrible vision I ever had was on the 8th of December last (1858). I woke up one morning before dawn, but, as mamma burns a lamp every night, it was quite light in our room. I had been awake about ten minutes or a quarter of an hour, and could not go to sleep, do what I would. However, as my mind was very pleasantly occupied, I did not mind much. Of a sudden I heard a heavy stamp, as if some one were trying to attract my attention by stamping with the foot. I raised my head, and to my horror saw an old person, who might have been a man or a woman; for the figure had on a white dressing-gown, and a kind of black skull or Glengariff cap. I could not see any hair, or should have been better able to judge of the sex. The face was that of a corpse, pinched and drawn by long illness and old age. The profile was turned towards me, and was delicate and regular, and clearly defined against the wall at the side of it. One hand was across the chest or waist, and the other hanging straight down. I rose on my elbow the better to make my observations. There were no clothes hanging in that part of the room, so that I could not have been deceived by anything of that kind. It stood by mamma's side, and as I gazed took three steps, each accompanied by a heavy stamp, and stopping at every step. I was perfectly calm while taking in all these particulars, but after the third step I was overcome by terror, as the figure was coming round my side; and clasping my little sister, as if even her tiny form would yield me protection, I prayed that the Almighty would remove the vision, and cause mamma to wake. I only heard one step after that. After a few minutes I determined to tie a knot in my handkerchief, under the pillow, as I knew mamma would say in the morning it was all a dream. Just as I was about to do this she woke. I spoke to her, and taking courage, looked at my watch, and found that it was about twenty minutes to six. I did not mention what I had seen till next day, or rather until it was light. I feel convinced that it was a forewarning of either my grandfather's or grandmother's death, as they have both been failing rapidly of late.

"I forgot to mention one case that happened before the last, and which should have had the precedence. One morning, in March, 1858, I was giving a lesson at Miss M.'s school here, and, looking up, I saw a thin man in blue cloth coat with turn-down velvet collar, standing by the side of my pupil. His figure was just like poor Mr. G., the violinist. His face I could not see, as my pupil's head came between us. I was startled and screamed, thinking it was one of the masters at the first glance. I just had time to notice it when it vanished. I told mamma when I got home. Next day we heard that poor G. had died at just about the time I saw his figure. I had not even heard that he was ill, and knew nothing of it till I was told he was dead.

"Another case I forgot to mention, occurred, I think, some time in last November. I was aroused from my sleep by a loud knocking at my bed-head. After I woke I listened, and in a few minutes heard it again. I said to mamma, 'Do you hear that?'—'Hear what, child?'—'Why, that loud knocking.' She said, 'Why, I have been awake for more than a quarter of an hour, and there has not been a sound that I could hear.' Afterwards I heard it again at the window. It was daylight, and I could see if there had been any one there; but I saw nothing. I told mamma I was sure we should hear of the death of some one we knew; and sure enough, a few weeks after, we heard that my aunt's father had been found dead in his room, just about the time I heard the knocking. I was a favourite of his when he was living.

"I cannot remember anything more now; I think I have mentioned every apparition that I have ever seen."

---

